# Species identification, phylogenetic analysis and detection of herbicide-resistant biotypes of *Amaranthus* based on ALS and ITS

**DOI:** 10.1038/s41598-020-68541-x

**Published:** 2020-07-16

**Authors:** Han Xu, Xubin Pan, Cong Wang, Yan Chen, Ke Chen, Shuifang Zhu, Rieks D. van Klinken

**Affiliations:** 1 Institute of Plant Quarantine, Chinese Academy of Inspection and Quarantine, Beijing, 100176 China; 2 CSIRO, GPO Box 2583, Brisbane, QLD 4001 Australia

**Keywords:** Taxonomy, Invasive species, Agroecology, Phylogenetics

## Abstract

The taxonomically challenging genus *Amaranthus* (Family Amaranthaceae) includes important agricultural weed species that are being spread globally as grain contaminants. We hypothesized that the ALS gene will help resolve these taxonomic challenges and identify potentially harmful resistant biotypes. We obtained 153 samples representing 26 species from three *Amaranthus* subgenera and included in that incorporated *ITS*, *ALS* (domains C, A and D) and *ALS* (domains B and E) sequences. Subgen. *Albersia* was well supported, but subgen. *Amaranthus* and subgen. *Acnida* were not. *Amaranthus tuberculatus*, *A. palmeri* and *A. spinosus* all showed different genetic structuring. Unique SNPs in ALS offered reliable diagnostics for most of the sampled *Amaranthus* species. Resistant ALS alleles were detected in sixteen *A. tuberculatus* samples (55.2%), eight *A. palmeri* (27.6%) and one *A. arenicola* (100%). These involved Ala_122_Asn, Pro_197_Ser/Thr/Ile, Trp_574_Leu, and Ser_653_Thr/Asn/Lys substitutions, with Ala_122_Asn, Pro_197_Thr/Ile and Ser_653_Lys being reported in *Amaranthus* for the first time*.* Moreover, different resistant mutations were present in different *A. tuberculatus* populations. In conclusion, the *ALS* gene is important for species identification, investigating population genetic diversity and understanding resistant evolution within the genus *Amaranthus*.

## Introduction

*Amaranthus* (Family Amaranthaceae) is a cosmopolitan genus with at least 70 species, including ancient cultivated plants such as the grains *Amaranthus cruentus*, *A. caudatus* and *A. hypochondriacus*, the leafy vegetable and ornamental *A. tricolor*, and the well-known invasive plant *A. retroflexus*^1,2^. In addition, the important agricultural weeds *A. palmeri* and *A. tuberculatus* are developing herbicide-resistant biotypes, which in turn can spread globally as seed contaminants in grain^3,4^. *Amaranthus* species can be difficult to identify, generally requiring adult plants. Genetic tools are urgently needed to rapidly identify *Amaranthus* samples at any life stage, and to recognize the emergence or entry of new herbicide-resistant biotypes.

In classical taxonomy, *Amaranthus* is divided into roughly three subgenera^5^, but these aren’t fully supported by genetic studies. Two subgenera, *Amaranthus* subgen. *Acnida* (L.) Aellen ex K.R. Robertson and *Amaranthus* subgen. *Albersia* (Kunth) Gren. & Godr., are not monophyletic groups based on the analysis of nuclear, chloroplast genes and genomes^6,7^. Phyletic position of some species varies depending on which gene is used. The subgeneric classifications of *A. palmeri*, *A. dubius* and *A. spinosus*, *A. tuberculatus* and *A. arenicola*, requires further clarification.

*Amaranthus palmeri* and *A. tuberculatus* have both evolved herbicide resistant (R) biotypes to at least four herbicide groups: those that target acetolactate synthase (ALS), photosystem II, protoporphyrinogen oxidase (PPO), and 5-enolpyruvylshikimate-3-phosphate (EPSP) synthase^8^. This is resulting in considerable losses to agriculture, and the continued evolution of resistance poses a significant threat to global food security^9–11^. This is especially concerning as their seeds are often intercepted in abundance among imported grains, and the R biotypes are very likely to be introduced as seed contaminants into importing countries. Jasieniuk et al. considered that the initial frequency and inheritance of resistant alleles are the key factors contributing to the evolution and spread of herbicide-resistant weeds^12^. Genetic tools are therefore needed to both identify herbicide resistance and to detect the global movement of R alleles.

Acetolactate synthase (ALS) is nuclear encoded, produced in the cytoplasm and transported via a transit peptide to the chloroplasts^13^. One mechanism of ALS inhibitor resistance is target site-based resistance, which is due to a single nucleotide substitution in the ALS gene^14^. These mutations involve amino acid substitutions at Ala_122_, Pro_197_, Ala_205_, Asp_376_, Arg_377_, Trp_574_, Ser_653_, and Gly_654_^15^. Under herbicide selection pressure, the R ALS alleles are dominant over the susceptive (S)^16^. Therefore, the frequency of resistance occurrence to ALS inhibitors and mutation frequency of ALS should be high. *ALS* inhibitor resistance has been reported in 66 species globally^13^, including in six *Amaranthus* species^15^. The *ALS* gene has also served as a useful molecular marker to study *Amaranthus*^17^.

*ITS* sequences have been widely used to resolve phylogenetic issues at different taxonomic levels^18^, and are selected as candidate barcodes for plants^19^. Song et al. (2000) and Xu et al. (2017) constructed phylogenetic trees among 16 and 23 species (respectively) of *Amaranthus* in China based on *ITS*^20,21^. Murphy et al. (2017) successfully detected *A. palmeri* in mixed samples using a quantitative PCR method using *ITS*^22^. Waselkov et al. (2018) analyzed the phylogenetic relationships of 58 species in *Amaranthus* based on *ITS* and a further five genes^7^. Murphy and Tranel (2018) used species-specific SNPs within the *ITS* region to identify and validate *Amaranthus* spp.^23^. All of these studies show that *ITS* is useful for identifying *Amaranthus* species, although limitations remain.

In this study we chose the ALS and ITS gene regions as molecular markers to analyze the phylogeny of *Amaranthus*, reexamine the taxonomic status of species encountered in China and to find effective SNPs for identifying species that are difficult to distinguish morphologically. Furthermore, we tested for the presence of ALR R alleles in China, with a special focus on samples obtained from grain imports and of naturalized *Amaranthus* populations in and around port facilities. This is a first step towards quantifying the global movement of herbicide-resistant alleles through contaminants in the global grain trade.

## Results

### Sequencing analysis

125 sequences of *ITS*, 85 sequences of *ALS* (domains C, A and D) and 78 sequences of *ALS* (domains B and E) in *Amaranthus* were generated for this study (Table S1). Both ALS regions were successfully amplified in 65 samples (16 *Amaranthus* species). These two partitioned regions were combined for phylogenetic analysis. All positions containing gaps and missing data were eliminated, and both ends of primers were cut off. There were a total of 621-nt to 624-nt of *ITS*, 429-nt of *ALS* (domains C, A and D), 604-nt to 605-nt of *ALS* (domains B and E) in the final data set. The ITS sequences had 67 variable sites and 61 Parsimony-Informative Sites (PIS), ALS (domains C, A and D) had 28 variable sites and 24 PIS and ALS (domains B and E) had 48 variable sites and 33 PIS.

### Phylogenetic analysis

**Figure 1:**
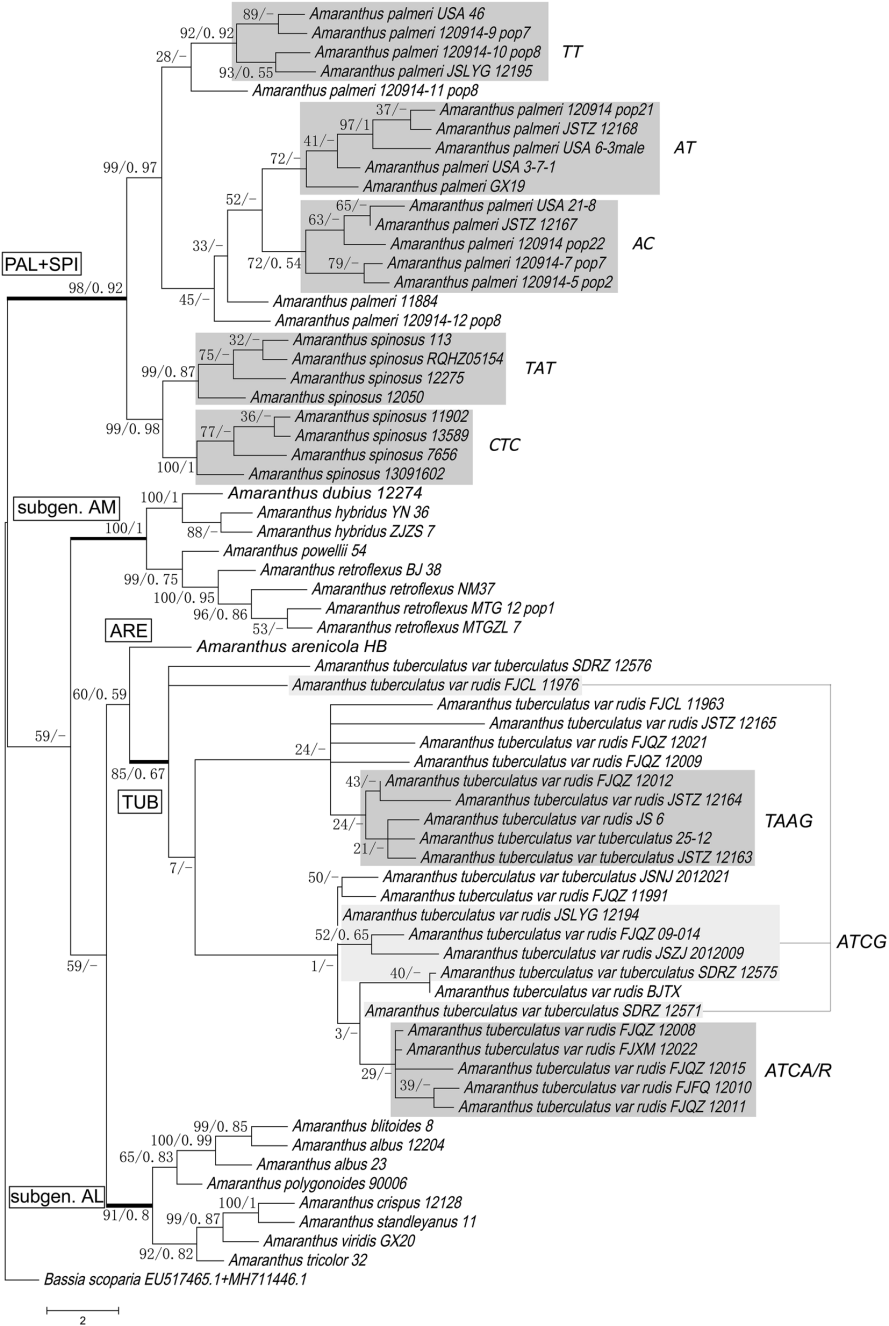
A maximum likelihood combined gene tree based on ITS and two regions within ALS of *Amaranthus* and one outgroup. Values at each node indicate maximum likelihood bootstrap support (BS)/Bayesian inference posterior probability (PP) value. Individuals marked with grey backgrounds represent genetically distinct groupings.

**Figure 2:**
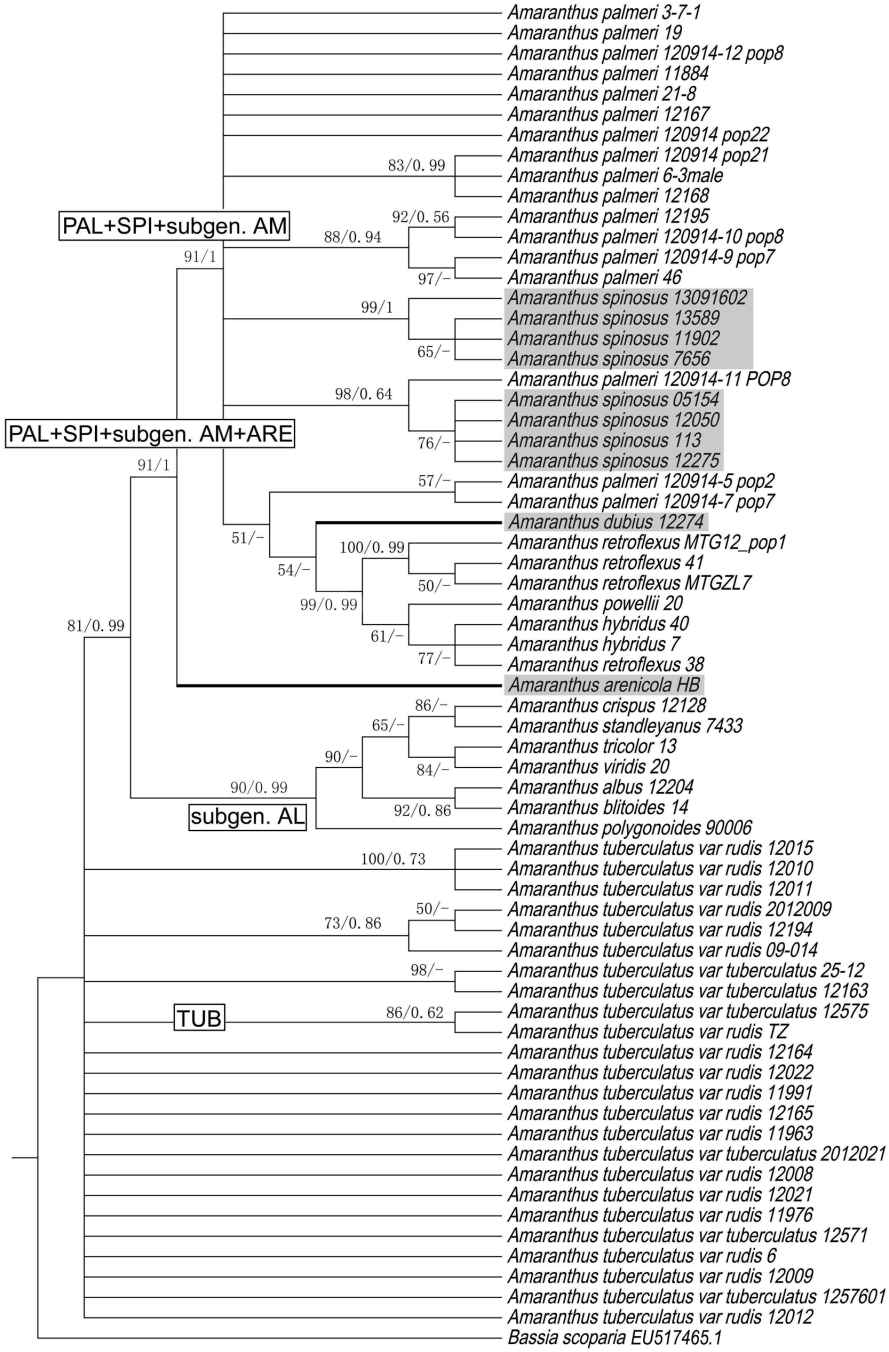
Maximum likelihood gene trees based on two regions within ALS. *Amaranthus* clades are delimited with grey highlight indicating anomalous samples (see text). Values at each node indicate maximum likelihood bootstrap support (BS)/Bayesian inference posterior probability (PP) value. The branches of *A. albus*, *A. blitoides*, *A. blitum*, *A. palmeri*, *A. spinosus* and parts of *A. tuberculatus* were compressed.

A total of 126 ITS and 66 ALS sequences from 27 *Amaranthus* species and one outgroup (*Bassia scoparia*, Genbank accessions: EU517465.1, MH711446.1) were used to construct the phylogenetic trees. According to genetic tree produced by combining ITS and ALS, four clades can be differentiated. *Amaranthus palmeri* and *A. spinosus* grouped together in one clade (PAL + SPI) (BS/PP = 98/0.92), *A. tuberculatus* and *A. arenicola* grouped together in another (TUB + ARE) (BS/PP = 60/0.59), and species in subgen. *Amaranthus* (AM) (BS/PP = 100/1) and subgen. *Albersia* (AL) (BS/PP = 91/0.8) formed their own clades (Fig. 1). In the ITS tree, except *A. arenicola* clustered with *A. tuberculatus* closely (BS/PP = 96/1), and other branches were maintained basically besides BS/PP value were decreased slightly (Fig. S1). These clades did not completely align with those produced in the ALS tree. *Amaranthus palmeri*, *A. spinosis* and subgen. *Amaranthus* formed a monophyletic clade (BS/PP = 91/1), with two branches of *A. spinosus* (BS/PP = 99/1; BS/PP = 76/-), *A. dubius* (subgen. *Amaranthus*) and the rest of subgen. *Amaranthus* (BS/PP = 99/0.99) nested within *A. palmeri*. Subgen. *Albersia* again formed a single clade. *Amaranthus arenicola* (ARE) was no longer clustered with *A. tuberculatus*, instead becoming a basal clade to *A. palmeri*, *A. spinosus*, *A. dubius* and subgen. *Amaranthus* (BS/PP = 99/1) (Fig. 1). *Amaranthus dubius* was not aggregated with subgen. *Amaranthus* any more (BS/PP = 54/-) (Fig. 2).

On the phylogenetic tree produced by combining ITS and ALS sequences, *A. palmeri*, *A. spinosus* and *A. tuberculatus* showed considerable intraspecific variation and structuring, especially *A. tuberculatus*. The *A. tuberculatus* clade can be subdivided into multiple groups according to the mutations located between loci 1,770 and 1794 on the *ALS* gene. These included groups of homozygous individuals (ATCA, ATCG, and TAAG) and individuals with heterozygous mutations at these loci or with irregular point mutations at other loci (Fig. 1, Table 1). *Amaranthus palmeri* was split up into three sections based on two mutations on the *ALS* gene (Fig. 1, Table 1). *Amaranthus spinosus* branched out into two groups by three mutations on the *ALS* (Fig. 1, Table 1)*.*

**Table 1:** Comparison of SNPs between the similar species: *Amaranthus tuberculatus*, *A. arenicola*, *A. palmeri*, *A. spinosus*, *A. dubius* and *A. hybridus* on ALS (domains C, A and D) and ALS (domains B and E).

Column heading		*Amaranthus* species (number of samples)					
ALS domains	Base positions	*A. tuberculatus (24)*	*A.arenicola (2)*	*A. palmeri (17)*	*A. spinosus (8)*	*A. dubius (1)*	*A. hybridus (2)*
ALS (domains C, A and D)	351	T	A	A	A	A	A
	363	C	C	C	C	Y	T
	465	T	C	C	C	C	C
	495	G/S1*	A/R	A/R5*	A	A	A
	513	T	T/C	T/C3*/Y8*	T	T	T
	516	C	T	T	T	T	T
	520	T	C	C	C	C	C
	531	A	A/R	A/R7*/G2*	A	A	A
	549	G	G	G/T1*/K2*	G	K	T
	552	T	T/Y	T/C3*/Y6*	T	T	T
	574	C/M2*	Y/M	C/Y2*/M1*	C	C	C
	575	C	C/Y	C	C	C	C
	576	T	C	C	C	C	C
	579	G/A3*/R3*	G	G	G	G	G
	603	T	T/Y	T/C3*/Y6*	T	T	T
	615	T	A	A	A	A	A
	636	A	C	C	C	C	C
	642	T	T	T/C1*/Y1*	T	Y	C
	678	C	T	T	T	T	T
ALS (domains B and E)	1,435	A	A	G	G	G	G
	1,482	C8*/T4*/Y12*	T	C	C	C	C
	1503	C23*/M1*	C	C/T1*/Y1*	C5*/T4*	C	C
	1551	A	A	A14*/W3*	A4*/T5*	W	A
	1554	C	C	T16*/Y1*	T	T	T
	1587	G17*/A1*/R6*	R	G16*/R1*	G	G	G
	1602	T23*/Y1*	T	T15*/Y2*	C5*/T4*	T	T
	1611	T6*/C7*/Y11*	Y	T	T	T	T
	1656	G/R6	R	G	G	R	A
	1,770	T6*/A11*/W7*	W	T	T	T	T
	1776	T11*/W8*/A5*	W	T	T	T	T
	1782	C11*/M8*/A5*	M	C	C	C	C
	1794	G20*/A3*/R2*	G	G	G	G	G
	1809	T	T	C13*/Y4*	C	T	T
	1836	C	C	T14*/Y2*/C1*	T	C	C
	1899	A	A	G	G	R	G
	1955	G19*/R4*/C1*	R	G	G	G	G
	1956	C17*/Y5*/S1*/T1*	Y	C16*/T1*	C	C	C
	1974	C18*/T1*/Y5*	Y	C15*/Y2*	C	C	C
	1980	C	C	C	C	M	A
	1983	A	A	A12*/T4*/W1*	A	A	A
	2016	T	T	T11*/C5*/Y1*	T	C	T

*The number of this kind of base on the locus. Underlines indicate mutations that separate *Amaranthus tuberculatus*, *A. spinosus* and *A. palmeri* into different groups.

### Interspecific and intraspecific variations on ITS and ALS

The average interspecific and intraspecific patristic distances were 0.02582 and 0.00054 respectively for the 125 ITS sequences, and 0.0155 and 0.00162 for the 65 ALS sequences (including the two partitioned regions). ITS was conserved relative to the ALS gene. Intraspecific variation frequency in ALS was greatest for *A. tuberculatus* (5.13%), *A. palmeri* (4.81%), and *A. spinosus* (0.75%), much higher than observed in ITS (0.32%, 0.16%, and 0.00% respectively). Of the 24 *A. tuberculatus* samples for which both ALS regions were sequenced, there were 22 PIS on ALS*,* but only one SNP on ITS.

*Amaranthus arenicola* had similar ALS (domains C, A and D) genotypes to *A. palmeri* and similar ALS (domains B and E) and identical ITS genotypes to *A. tuberculatus* (Table 1). *Amaranthus dubius* (subgen. *Amaranthus*) had six heterozygotes across the two ALS regions, with half of the alleles of six heterozygotes being identical to those of *A. spinosus*, and the remainder being identical to the rest of subgen. *Amaranthus* (Table 1). Three stable mutations of *A. spinosus* in *ALS* resulted in all of them grouping into two sections (Fig. 1, Table 1), despite these individuals only having one SNP in ITS. Meanwhile, on the above same loci, several *A. palmeri* also had corresponding heterozygotes (Table 1), and one individual was almost consistent with *A. spinosus* with respect to two regions of ALS except for six heterozygotes (Fig. 2). In addition, there were one to two SNPs in *A. albus* and *A. retroflexus*, 6 indels in *A. blitum* on ITS, and two SNPs in *A. retroflexus* on ALS. *Amaranthus standleyanus* and *A. crispus* only differed by one base. *Amaranthus capensis* differed from *A. tenuifolius* and *A. tricolor* by one SNP in ITS.

### R *ALS* alleles in *A. arenicola, A. palmeri* and *A. tuberculatus*

In our analysis of *ALS*, 25 plants with R *ALS* alleles were detected. Sixteen *A. tuberculatus* (55.2% of samples), eight *A. palmeri* (27.6%) and one *A. arenicola* (100%) were detected with Ala_122_Asn, Pro_197_Ser/Thr/Ile, Trp_574_Leu, and Ser_653_Thr/Asn/Lys substitutions (Table 2). Of the 31 amino acid mutations of 25 R biotypes, 77.4% were heterozygous. The Ala_122_Asn mutations were mainly generated in Fujian’s population of *A. tuberculatus*. However, Ser_653_Thr/Asn/Lys mutations were found in *A. tuberculatus* populations from Jiangsu and Shandong (Table 2). The major resistant substitutions in *A. palmeri* were Pro_197_Ser/Thr and Trp_574_Leu: Acession No. 7229 sample had homozygous mutations of Trp_574_Leu, while the remaining mutations were heterozygous (Table 2). Five individuals of *A. tuberculatus* and one *A. arenicola* had two amino acid substitutions (Table 2). The amino acid substitutions of *A. arenicola* mostly occurred in Pro_197_Ile and Ser_653_Thr (Table 2). Among them, the substitutions of Ala_122_Asn, Pro_197_Thr/Ile and Ser_653_Lys were the first to be observed in *Amaranthus.*

**Table 2:** Amino acid substituts in sampled species with *R* ALS alleles^*^.

Species	Origin	Accession no	Amino acid substitutions			
			Ala_122_Asn (G_349_A/R; C_350_A/M)	Pro_197_Ser/Thr/Ile (C_574_Y/M;C_575_Y)	Trp_574_Leu (G_1718_T/K)	Ser_653_Thr/Asn/Lys (G_1955_C/R/S; C_1956_S/Y)
*Amaranthus palmeri*	Beijing	120914_7 pop7	GCA	CCC	T*KG	AGC
*A. palmeri*	Beijing	7229	GCA	CCC	T*TG	AGC
*A. palmeri*	Beijing	120914_11 pop8	GCA	*YCC	TGG	AGC
*A. palmeri*	Beijing	120914_12 pop8	GCA	*YCC	TGG	AGC
*A. palmeri*	Beijing	120914_16 pop10	GCA	*YCC	TGG	AGC
*A. palmeri*	Guangxi	19	GCA	CCC	T*KG	AGC
*A. palmeri*	Hebei	180910	GCA	*YCC	TGG	AGC
*A. palmeri*	Jiangsu	12167	GCA	*MCC	TGG	AGC
*A. tuberculatus var. rudis*	Fujian	11963	GCT	CCT	T*KG	AGY
*A. tuberculatus var. rudis*	Fujian	12010	*A*AT	CCT	TGG	AGC
*A. tuberculatus var. rudis*	Fujian	12008	*R*MT	CCT	T*KG	AGC
*A. tuberculatus var. rudis*	Fujian	12011	*A*AT	CCT	TGG	AGC
*A. tuberculatus var. rudis*	Fujian	12012	GCT	CCT	T*KG	AGT
*A. tuberculatus var. rudis*	Fujian	12015	*A*AT	CCT	TGG	AGC
*A. tuberculatus var. rudis*	Fujian	09–014	GCT	*MCT	TGG	AGC
*A. tuberculatus var. rudis*	Fujian	12022	*R*MT	CCT	T*KG	AGC
*A. tuberculatus var. rudis*	Jiangsu	6	GCT	CCT	TGG	A*R*S
*A. tuberculatus var. rudis*	Jiangsu	12194	GCT	CCT	T*KG	A*R*Y
*A. tuberculatus var. rudis*	Jiangsu	12165	GCT	*MCT	T*KG	AGC
*A. tuberculatus var. rudis*	Jiangsu	2012009	GCT	CCT	TGG	A*CC
*A. tuberculatus* var. *tuberculatus*	Jiangsu	2012021	GCT	CCT	TGG	A*RC
*A. tuberculatus* var. *tuberculatus*	Shandong	12571	GCT	CCT	T*TG	AGC
*A. tuberculatus* var. *tuberculatus*	Shandong	12575	GCT	CCT	T*KG	A*RC
*A. tuberculatus* var. *tuberculatus*	Shandong	12576	GCT	CCT	T*KG	AGC
*A. arenicola*	Hebei	HB	GCA	*M*YC	TGG	A*SY

## Discussion

Our research demonstrates the considerable value of ALS for clarifying phylogenetic relationships within the genus *Amaranthus*, in assisting with the identification of species that are morphologically difficult to distinguish, and for identifying the presence of herbicide-resistant genes. The main findings and their significance are discussed below.

Our phylogenetic analysis supported subgen. *Albersia* as a single clade, but the boundary between subgen. *Amaranthus* and subgen. *Acnida* was confused by *A. palmeri*, *A. spinosus*, *A.arenicola* and *A. dubius*. Previous morphological and molecular studies have found *A. spinosus* and *A. palmeri* to be closely related^24^. For example, Riggns et al. (2010) classified *A. spinosus* as a sister group of *A. palmeri* based on the *ALS* gene^17^.The sequence homology of EPSP synthase (EPSPS) between glyphosate-resistant *A. spinosus* and glyphosate-resistant *A. palmeri* supported the hypothesis that the EPSPS amplicon in *A. spinosus* originated from *A. palmeri*^25^. We have observed a similar situation in the ALS gene, namely *A. spinosus* was grouped into two sections by three stable mutations in *ALS* and formed parallel branches with *A. palmeri*. Furthermore, several *A. palmeri* plants growing together with *A. spinosus* also had corresponding heterozygotes at the above loci, one of which was consistent with *A. spinosus* with respect to two regions of ALS except for six heterozygotes. The other sampled *A. spinosus* individuals came from different regions, grew alone, and have naturalized in inner provinces. Although *A. spinosus* and *A. palmeri* have many opportunities to implement gene exchange in nature^26^, it seems that these intraspecific mutations in *A. spinosus* have been formed for a long time, and were not an occasional or transient result of hybridization.

*A. arenicola* is a dioecious species. Female flowers have five perianths like *A. palmeri* and other species in subgen. *Amaranthus*, whereas its’ short bracts and long spikes are similar to *A. tuberculatus*. Stetter and Schmid (2017) placed *A. arenicola* and *A. palmeri* together based on genotyping by sequencing methods^6^. In contrast, Waselkov et al. (2018) concluded from their analysis of four nuclear genes and two chloroplast sequences that *A. arenicola* is closely related to *A. tuberculatus*, and believed that the samples of *A. arenicola* used by Stetter and Schmid (2017) were wrongly identified^6,7^. We also found that *A. arenicola* was closer to *A. palmeri* than *A. tuberculatus* based on *ALS* (domain C, A and D), while on *ITS* and *ALS* (domain B and E), *A. arenicola* was basically within the variation range of *A. tuberculatus*. This discovery is very interesting as we observed *A. arenicola, A. tuberculatus*, *A. palmeri* and *A. hybridus* growing together in the port monitoring area with many heterozygous mutations at some loci being shared among them. The significance of this finding warrants further study.

*Amaranthus tuberculatus* was previously considered to be two largely allopatric species, *A. tuberculatus and A. rudis*, according whether the fruit is dehiscent or not, the number of perianth segments, and different geographical origins^27^. However, a large number of morphological intermediate forms exist between the two, and they are now treated as one species^27^. Riggins et al. (2010) reported heterozygous mutations on *ALS* for six individuals of *A. palmeri* and *A. tuberculatus*^17^. In our study, high SNP diversity in ALS resulted in considerable genotypic structuring within *A. tuberculatus*, but it did not correspond with our morphological sub-species identifications. Waselkov et al. (2018) speculated that present-day *A. tuberculatus* was derived from hybridization of two ancestors originating from different dioecious species^7^. Combined with our finding, *A. arenicola* has homologous mutation types at the same loci as *A. tuberculatus* at ALS (domain B and E) and ITS. In the place of origin (North America), the male plants of *A. arenicola* are often incorrectly identified as *A. tuberculatus*^28^. We speculated that in some cases the male plants of *A. tuberculatus* might male with the females of *A. arenicola* when it entered into a new habitat where is absent of conspecific male plant.

*Amaranthus dubius* is a known allotetraploid that originated through hybridization between *A. spinosus* and other species in the *A. hybridus* aggregate^29^. Waselkov et al. (2018) found that *A. dubius* is strongly supported as the sister species to *A. spinosus* in the chloroplast tree, but clustered with *A. hybridus* aggregates in the nuclear tree^7^. In our study, the phylogenetic position of *A. dubius* on *ALS* was an out-group to the remaining species in the subgen. *Amaranthus*. This was supported by the results from SNP and AFLP Package analysis using biallelic markers^6^. We can deduce from the heterozygote loci that *A. dubius* has a hybrid origin^7^.

Herbicide resistance already poses significant challenges for managing *Amaranthus* weeds in its native range. We detected Ala_122_Asn, Pro_197_Thr/Ile and Ser_653_Lys substitutions on the ALS gene for the first time in *Amaranthus*. According to the website International Survey of Herbicide Resistance^8^, the Ala_122_Asn has previously only been reported on *Echinochloa crus-galli* var. *crus-galli* in 2017 where it resulted in strong resistance to four kinds of herbicides^15^. The substitution of Pro_197_Thr is relatively common and has been reported on numerous species, whereas the substitution of Pro_197_Ile had only previously been reported on *Sisymbrium orientale*^15^. Both amino acid substitutions lead to herbicide resistance^15^. The newly discovered substitution Ser_653_Lys has not previously been reported on any species, and its significance remains unclear.

All of the R ALS alleles we detected were in plants sampled from border monitoring areas within China. These alleles are therefore most likely to have entered China as contaminants of imported grain. Up to 77.4% of the R ALS alleles were heterozygous. R *ALS* alleles are dominant over S alleles, so can still be selected even under heterozygous conditions^16^. This is the first study of R ALS alleles in *Amaranthus* within China, and little is known of their distribution globally beyond some studies in North America. Further work is needed to better understand the threat these and other alleles pose to agriculture in China, including how well these new R biotypes will spread and perform.

We can only make preliminary inferences from genes. Homozygous mutations of Trp_574_Leu were detected in a sample of *A. palmeri* coded No. 7229 taken from the Beijing population in 2004. This population was within a processing plant for imported grain. In 2012, two heterozygous mutation biotypes of Trp_574_Leu and three heterozygous mutation biotypes of Pro_197_Ser were detected in the samples collected within 1 km of No. 7229. This suggests that after nearly ten years of establishment, the R *ALS* alleles have spread in the population as heterozygotes. Further work is required to test this hypothesis.

## Conclusions

Through our comparative analysis of *ALS*, we found that *ALS* can provide many valuable SNPs for species identification, such as for difficult species, *A. arenicola* and *A. dubius*, even the different genotypes under species level. On the basis of SNPs, suitable restriction enzymes can be found, and optimal primers can be designed. Moreover, different herbicide-resistant biotypes belonging to different populations of *A. tuberculatus* shows that these populations have different origins. Through genetic testing, resistant biotypes can be found quickly. This will be critical for managing herbicide resistance in *Amaranthus* into the future.

## Materials and methods

### Plant materials and morphological identification

We collected 153 plant and seed samples from 26 species of *Amaranthus* between 2005 and 2018 (Table S1). 48 plant samples were taken from populations that appear to have been naturalized for some time (including one from Spain). 96 samples were from plants growing in or adjacent to ports, wharfs and processing plants of imported grain where imported grains may leak. These are most likely to have established from contaminated seed. Nine accessions were seeds intercepted from grains imported from the United States (8) and Canada (1). Most samples (n = 109) were plant samples collected from imported grain processing plants and wharfs located at ports within China. 35 plant samples were taken from fields, wastelands and lawns in China and one in Spain.

Seeds were sowed in a Beijing greenhouse (14–25 °C, humidity 50–70%) from April to May (2008). The resulting eight adult plants were harvested for further study. Identification relied on *Amaranthus* monographs^1,2,28,30^. One adult plant of *A. arenicola* originated from intercepted seeds was collected in a plant quarantine nursery. We also referenced the type specimen of *Amaranthus arenicola* I.M. Johnst. (Collector: A.S. Hitchcock, #428A, stored in Missouri Botanical Garden (MO), MO-247457) on the website of JSTOR Global Plants (www.jstor.org).

### DNA extraction and PCR

About 10 mg leaves of each individual of 153 samples were dried by Silica for DNA extraction. These dried leaves were ground into a powder by Grinding Mill (Retsch MM400, Germany) for 1 min (1,800 r·min^−1^), and DNA was extracted by Plant Genomic DNA Kit (Tiangen Biotech Co., China). *Amaranthus palmeri* S. Watson (*ITS* accession: KF493784.1, KC747433.1 and KP318856.1; *ALS* accession: KY781923.1) and *Amaranthus retroflexus* L. (*ALS* accession: AF363369.1) were used as resistant biotype references.

A pair of universal primers *ITS1* (5′-TCCGTAGGTGAACCTGCGG-3′) and *ITS4* (5′- TCCTCCGCTTATTGATATGC-3′) were used to amplify the ITS region*.* The PCR reaction mixture contained 1 μL of DNA template (approximately 30 ng of genomic DNA), 10 × of Ex Taq buffer without MgCl2, 10 μM each of forward and reverse primers, 2.5 mM of each dNTP, 2.5 mM MgCl_2_ and 1 U of ExTaq polymerase (Takara) with ddH_2_O for a final volume of 25 μL. PCR cycling conditions were: template denaturation at 94 °C for 5 min followed by 30 cycles of denaturation at 94 °C for 1 min, primer annealing at 55 °C for 1 min, and primer extension at 72 °C for 90 s, with a final extension of 10 min at 72 °C.

A pair of primers 2F (5′-GATGTYCTCGTYGARGCTCT-3′) and 2R (5′-AAYCAAAACAGGYCCAGGTC-3′) were used to amplify a region of the ALS gene containing nucleotides coding for Ala_122_ (conserved nucleotides in domain C), Pro_197_ (domain A), and Ala_205_ (domain D), all of which have been linked to *ALS* inhibitor resistance^14^. Another pair of primers 5F (5′-ATTCCTCCGCARTATGCSATT-3′) and 6R (5′-CCTACAAAAAGCTTCTCCTCTATAAG-3′) were used to amplify the region within the ALS encompassing domains B (comprising Ser_653_) and E (the carboxy terminal region, including 13 amino acids after the stop codon^14^). The PCR reaction mixture contained 1.3 μL of DNA template (approximately 30 ng of genomic DNA), 10 × of Ex Taq buffer without MgCl_2_, 10 μM each of forward and reverse primers, 2.5 mM of each dNTP, 2.5 mM MgCl_2_ and 1 U of ExTaq polymerase (Takara) with ddH_2_O for a final volume of 25 μL. PCR cycling conditions were: template denaturation at 94 °C for 10 min followed by 35 cycles of denaturation at 94 °C for 1 min, primer annealing at 57 °C for 80 s, and primer extension at 72 °C for 80 s, with a final extension of 10 min at 72 °C. Each PCR product was sequenced in forward and reverse directions to minimize sequencing errors.

### Phylogenetic analysis

The alignment and adjustment of multiple sequences and SNPs detection were carried out by MAFFT v7.450^31^ and Geneious Prime v 2020.1.2 (Biomatters, Auckland, New Zealand). The aligned coding sequences of *ITS* and *ALS* (domains C, A and D; domains B and E) were concatenated. The DNA substitution model (GTR + I + G model) was chosen using jModelTest 2.1.6^32^, and used in maximum likelihood (ML) analysis and Bayesian inference. ML analysis was conducted using RAxML version 8.0.0^33^ on the Geneious Prime v 2020.1.2 (Biomatters, Auckland, New Zealand). Bayesian inference was conducted using MrBayes 3.2.6^34^ with Ngen = 1,000,000, Samplefreq = 200, and Burninfrac = 0.25. *Bassia_scoparia* (GenBank Accession: MH711446.1 and EU517465.1) served as the outgroup.

## Supplementary information

Supplementary file1 (PDF 837 kb)

## Data Availability

The data generated and analyzed in this study are available from the authors on request.
