# Supplementary material for: Species identification, phylogenetic analysis and detection of herbicide-resistant biotypes of Amaranthus based on ALS and ITS
Source: Sci Rep. 2020 Jul 16;10:11735. doi: 10.1038/s41598-020-68541-x (PMC7366686; doi:10.1038/s41598-020-68541-x)
Supplement: Supplementary file 1 — Supplementary file1 (PDF 837 kb) [file 41598_2020_68541_MOESM1_ESM.pdf]

Species identification, phylogenetic analysis and detection of herbicide-resistant biotypes of *Amaranthus* based on ALS and ITS

Han Xu*1, Xubin Pan1, Cong Wang1, Yan Chen1, Ke Chen1, Shuifang Zhu1, Rieks D. van Klinken2

1Institute of Plant Quarantine, Chinese Academy of Inspection and Quarantine, Beijing 100176, China
2
*To whom correspondence should be addressed. E-mail: xuhangin@gmail.com

Supplementary table and figure legends:

Table S1: Plant materials and origins.

Figure S1: Maximum likelihood gene trees based on ITS. *Amaranthus* clades are delimited with grey highlight indicating anomalous samples (see text). Values at each node indicate maximum likelihood bootstrap support (BS)/Bayesian inference posterior probability (PP) value. The branches of *A. albus* , *A. blitoides* , *A. polygonoides* , *A. blitum* , *A. viridis* , *A. palmeri* , *A. spinosus* and parts of *A. tuberculatus* were compressed.

Table S1. Plant materials and origins

Sample code	Species	Date collected	Where sampled				Plant or seed	Individual plants		
			Country	*Border monitoring area	**Local habitat	Origin of imported seed		ITS	ALS (domains C, A and D)	ALS (domains B and E)
12204	*Amaranthus albus*	2011	China		Jiangsu		Plant	√	√	√
12250	*Amaranthus albus*	2012	China		Guangxi		Plant	√	√	
11824	*Amaranthus albus*	2009	China		Liaoning		Plant	√		
2373	*Amaranthus albus*	1981	China		Xinjiang		Plant	√		
4698	*Amaranthus albus*	1989	China		Beijing		Plant	√		√
11500	*Amaranthus albus*	2008	China		Shandong		Plant	√	√	
12332	*Amaranthus albus*	2012	China		Xinjiang		Plant	√		√
12540	*Amaranthus albus*	2012	China		Xinjiang		Plant	√		
12536	*Amaranthus albus*	2012	China		Xinjiang		Plant	√		
12155	*Amaranthus albus*	2011	China	Jiangsu			Plant		√	
23	*Amaranthus albus*	2010	China	Unknown			Plant		√	
12771	*Amaranthus albus*	2012	China	Hubei			Plant	√		
HB	*Amaranthus arenicola*	2018	China	Hebei		USA	Plant	√	√	√
JSTZ	*Amaranthus arenicola*	2017	China	Jiangsu		USA	Seed	√	√	
12336	*Amaranthus blitoides*	2012	China		Xinjiang		Plant	√		
12413	*Amaranthus blitoides*	2012	China		Xinjiang		Plant	√		
13501	*Amaranthus blitoides*	2012	China	Jiangsu			Plant	√		
7469	*Amaranthus blitoides*	2008	China		Beijing		Plant	√		
14	*Amaranthus blitoides*	2011	China		Inner Mongolia		Plant	√	√	√
2	*Amaranthus blitum*	2008	China		Beijing		Plant	√		
25	*Amaranthus blitum*	2008	China		Tianjing		Plant	√		
37BJ	*Amaranthus blitum*	2008	China		Beijing		Plant	√		
7465	*Amaranthus bouchonii*	2008	China		Beijing		Plant	√		
12182	*Amaranthus capensis*	2011	China	Jiangsu			Plant	√		
4452	*Amaranthus caudatus*	2008	China		Beijing		Plant	√		
12128	*Amaranthus crispus*	2010	China	Hebei			Plant	√	√	√
4705	*Amaranthus cruentus*	2008	China		Beijing		Plant	√		
14	*Amaranthus cruentus*	Unknown	China		Guangxi		Plant			√

2011	*Amaranthus deflexus*	2012	Spain		Spain		Plant	√		
12274	*Amaranthus dubius*	2010	China	Shandong			Plant	√	√	√
ZJ	*Amaranthus fimbriatus*	2010	China	Zhejiang			Plant	√		
12204	*Amaranthus graecizans ssp. sylvestris*	2012	China		Xinjiang		Plant	√		
7	*Amaranthus hybridus*	2008	China	Zhejiang			Plant	√	√	√
40	*Amaranthus hybridus*	2008	China		Yunnan		Plant	√	√	√
120914-17pop1	*Amaranthus hybridus*	2012	China		Beijing		Plant		√	
120914-18pop1	*Amaranthus hybridus*	2012	China		Beijing		Plant		√	√
120914-19pop1	*Amaranthus hybridus*	2012	China		Beijing		Plant			√
5154	*Amaranthus hypochondriacus*	2008	China		Beijing		Plant	√		
1	*Amaranthus palmeri*	2008	China	Beijing			Plant	√		
14	*Amaranthus palmeri*	2008	China	Fujian			Plant	√		
19	*Amaranthus palmeri*	2008	China	Guangxi			Plant	√	√	√
24	*Amaranthus palmeri*	2008	USA			USA	Seed	√		
26	*Amaranthus palmeri*	2008	China	Beijing			Plant	√		
28	*Amaranthus palmeri*	2008	China	Beijing			Plant	√		
30	*Amaranthus palmeri*	2008	China	Beijing			Plant	√		
34	*Amaranthus palmeri*	2008	China	Beijing			Plant	√		
43	*Amaranthus palmeri*	2008	China			USA	Seed	√		
45	*Amaranthus palmeri*	2008	China	Beijing			Plant	√		
46	*Amaranthus palmeri*	2008	China	Beijing		USA	Seed	√	√	√
7185	*Amaranthus palmeri*	2004	China	Beijing			Plant	√		
7229	*Amaranthus palmeri*	2004	China	Beijing			Plant	√		√
11429	*Amaranthus palmeri*	2008	China	Tianjin			Plant	√		
11884	*Amaranthus palmeri*	2009	China	Beijing			Plant		√	√
11906	*Amaranthus palmeri*	2009	China	Beijing			Plant		√	
11907	*Amaranthus palmeri*	2009	China	Beijing			Plant		√	
11909	*Amaranthus palmeri*	2009	China	Beijing			Plant		√	
11911	*Amaranthus palmeri*	2009	China	Beijing			Plant		√	
11914	*Amaranthus palmeri*	2009	China	Beijing			Plant		√	
11964	*Amaranthus palmeri*	2010	China	Fujian			Plant	√		
11972	*Amaranthus palmeri*	2010	China	Fujian			Plant	√		
12165	*Amaranthus palmeri*	2011	China	Jiangsu			Plant	√		
12167	*Amaranthus palmeri*	2011	China	Jiangsu			Plant		√	√
12168	*Amaranthus palmeri*	2011	China	Jiangsu			Plant	√	√	√
12195	*Amaranthus palmeri*	2012	China	Jiangsu			Plant		√	√

12259	*Amaranthus palmeri*	2012	China	Guangxi			Plant	√		
12568	*Amaranthus palmeri*	2012	China	Shandong			Plant		√	
180910	*Amaranthus palmeri*	2018	China	Hebei			Plant	√	√	
11091301	*Amaranthus palmeri*	2011	China	Beijing			Plant	√		
11091302	*Amaranthus palmeri*	2011	China	Beijing			Plant	√	√	
120914_1 pop1	*Amaranthus palmeri*	2012	China	Beijing			Plant		√	
120914_10 pop8	*Amaranthus palmeri*	2012	China	Beijing			Plant	√	√	√
120914_11 pop8	*Amaranthus palmeri*	2012	China	Beijing			Plant	√	√	√
120914_12 pop8	*Amaranthus palmeri*	2012	China	Beijing			Plant		√	√
120914_16 pop10	*Amaranthus palmeri*	2012	China	Beijing			Plant	√	√	
120914_1	*Amaranthus palmeri*	2012	China	Beijing			Plant		√	
120914_pop21	*Amaranthus palmeri*	2012	China	Beijing			Plant	√	√	√
120914_pop22	*Amaranthus palmeri*	2012	China	Beijing			Plant	√	√	√
120914_4 pop1	*Amaranthus palmeri*	2012	China	Beijing			Plant		√	
120914_5 pop2	*Amaranthus palmeri*	2012	China	Beijing			Plant	√	√	√
120914_7 pop7	*Amaranthus palmeri*	2012	China	Beijing			Plant	√	√	√
120914_8 pop7	*Amaranthus palmeri*	2012	China	Beijing			Plant	√		√
120914_9 pop7	*Amaranthus palmeri*	2012	China	Beijing			Plant		√	√
13_6_1	*Amaranthus palmeri*	2008	China			USA	Seed	√	√	
21_8	*Amaranthus palmeri*	2008	China			USA	Seed	√	√	√
3_7_1	*Amaranthus palmeri*	2008	China			USA	Seed	√	√	√
5_14	*Amaranthus palmeri*	2008	China	Fujian			Plant			√
6_3male	*Amaranthus palmeri*	2008	China			USA	Seed	√	√	√
90006	*Amaranthus polygonoides*	2014	China		Beijing		Plant	√	√	√
ZJ	*Amaranthus polygonoides*	2012	China		Zhejing		Plant	√		
20	*Amaranthus powellii*	2006	China		Beijing		Plant	√	√	√
41	*Amaranthus retroflexus*	2008	China		Inner Mongolia		Plant	√	√	√
8	*Amaranthus retroflexus*	2008	China		Beijing		Plant	√	√	
7471	*Amaranthus retroflexus*	2006	China		Beijing		Plant	√		
MTG12_pop1	*Amaranthus retroflexus*	2012	China		Beijing		Plant		√	√
MTGZL7	*Amaranthus retroflexus*	2012	China		Beijing		Plant		√	√
38	*Amaranthus retroflexus*	2012	China		Beijing		Plant			√
42	*Amaranthus retroflexus*	2008	China		Beijing		Plant	√		
44	*Amaranthus retroflexus*	2008	China			Canada	Seed	√		
13589	*Amaranthus spinosus*	2012	China	Beijing			Plant	√	√	√
11902	*Amaranthus spinosus*	2009	China	Beijing			Plant	√	√	√
13091602	*Amaranthus spinosus*	2013	China	Beijing			Plant	√	√	√

7656	*Amaranthus spinosus*	2006	China	Beijing			Plant	√	√	√
12275	*Amaranthus spinosus*	2012	China		Guangxi		Plant	√	√	√
05154	*Amaranthus spinosus*	2005	China		RQHZ		Plant	√	√	√
12050	*Amaranthus spinosus*	2010	China	Fujian			Plant	√	√	√
113	*Amaranthus spinosus*	2008	China		Beijing		Plant	√	√	√
4756	*Amaranthus spinosus*	2006	China		Beijing		Plant	√	√	√
7433	*Amaranthus standleyansus*	2005	China	Zhejing			Plant	√	√	√
7464	*Amaranthus standleyansus*	2005	China	Zhejing			Plant	√		
JSTZ	*Amaranthus standleyansus*	2016	China	Jiangsu			Plant	√		
16	*Amaranthus tennuifolius*	2008	China		Shandong		Plant	√		
13	*Amaranthus tricolor*	Unknown	China		Beijing		Plant	√	√	√
TZ	*Amaranthus tuberculatus var.*	2011	China	Beijing			Plant	√	√	√
31	*Amaranthus tuberculatus var.*	2010	China	Fujian			Plant			√
11963	*Amaranthus tuberculatus var.*	2010	China	Fujian			Plant	√	√	√
11976	*Amaranthus tuberculatus var.*	2010	China	Fujian			Plant	√	√	√
11991	*Amaranthus tuberculatus var.*	2010	China	Fujian			Plant	√	√	√
12010	*Amaranthus tuberculatus var.*	2010	China	Fujian			Plant	√	√	√
12008	*Amaranthus tuberculatus var.*	2010	China	Fujian			Plant	√	√	√
12009	*Amaranthus tuberculatus var.*	2010	China	Fujian			Plant	√	√	√
12011	*Amaranthus tuberculatus var.*	2010	China	Fujian			Plant	√	√	√
12012	*Amaranthus tuberculatus var.*	2010	China	Fujian			Plant	√	√	√
12015	*Amaranthus tuberculatus var.*	2010	China	Fujian			Plant	√	√	√
12021	*Amaranthus tuberculatus var.*	2010	China	Fujian			Plant	√	√	√
09-014	*Amaranthus tuberculatus var.*	2009	China	Fujian			Plant	√	√	√
12022	*Amaranthus tuberculatus var.*	2010	China	Fujian			Plant	√	√	√
6	*Amaranthus tuberculatus var.*	2011	China	Jiangsu			Plant		√	√
12194	*Amaranthus tuberculatus var.*	2010	China	Jiangsu			Plant	√	√	√
12164	*Amaranthus tuberculatus var.*	2010	China	Jiangsu			Plant	√	√	√
12165	*Amaranthus tuberculatus var.*	2010	China	Jiangsu			Plant		√	√
2012009	*Amaranthus tuberculatus var.*	2012	China	Jiangsu			Plant	√	√	√
12571	*Amaranthus tuberculatus var.*	2011	China	Shandong			Plant	√	√	√
CQ	*Amaranthus tuberculatus var.*	2012	China	Chongqing			Plant	√		
11975	*Amaranthus tuberculatus var.*	2010	China	Fujian			Plant	√		
12014	*Amaranthus tuberculatus var.*	2010	China	Fujian			Plant	√		
36	*Amaranthus tuberculatus var.*	2010	China	Guangdong			Plant	√		
XS	*Amaranthus tuberculatus var.*	2014	China	Guangdong			Plant	√		
JS	*Amaranthus tuberculatus var.*	2014	China	Jiangsu			Plant	√		

5	*Amaranthus tuberculatus var.*	2010	China	Fujian			Plant	√		
11994	*Amaranthus tuberculatus var. tuberculatus*	2010	China	Fujian			Plant	√		
2012021	*Amaranthus tuberculatus var. tuberculatus*	2010	China	Jiangsu			Plant	√	√	√
12163	*Amaranthus tuberculatus var. tuberculatus*	2010	China	Jiangsu			Plant	√	√	√
12575	*Amaranthus tuberculatus var. tuberculatus*	2010	China	Shandong			Plant	√	√	√
12576	*Amaranthus tuberculatus var. tuberculatus*	2010	China	Shandong			Plant	√	√	√
12	*Amaranthus tuberculatus var. tuberculatus*	2010	China	Fujian			Plant	√		
11997	*Amaranthus tuberculatus var. tuberculatus*	2010	China	Fujian			Plant	√		
12019	*Amaranthus tuberculatus var. tuberculatus*	2010	China	Fujian			Plant	√		√
25-12	*Amaranthus tuberculatus var. tuberculatus*	2010	China	Fujian			Plant		√	√
20	*Amaranthus viridis*	2011	China		Guangxi		Plant	√	√	√
3	*Amaranthus viridis*	2008	China		Beijing		Plant	√		
39	*Amaranthus viridis*	2008	China		Beijing		Plant	√		
32	*Amaranthus viridis*	2010	China		Fujian		Plant	√		
33	*Amaranthus viridis*	2010	China		Fujian		Plant	√		

*Border monitoring area includes ports, wharfs, processing plants of imported grains and their around regions where imported grains maybe leak.

**Local habitat means naturalized places of alien species have colonized for a long time.

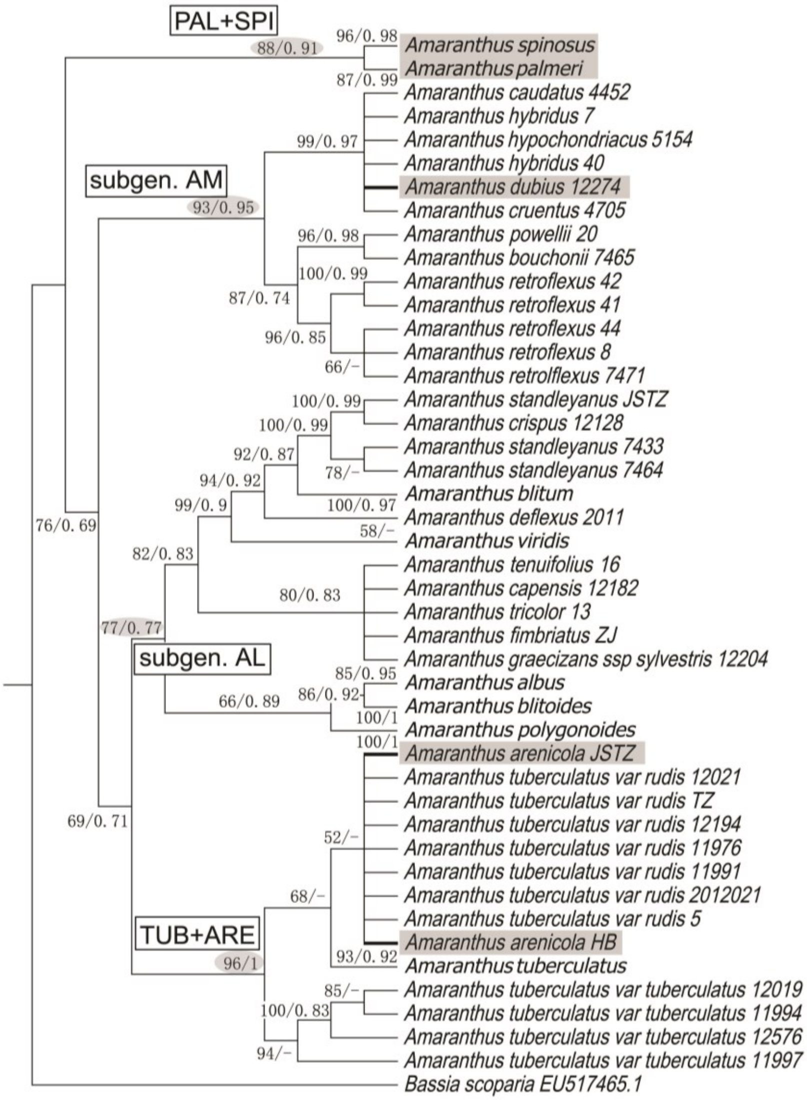

Figure S1: Maximum likelihood gene trees based on ITS. *Amaranthus* clades are delimited with grey highlight indicating anomalous samples (see text). Values at each node indicate maximum likelihood bootstrap support (BS)/Bayesian inference posterior probability (PP) value. The branches of *A. albus, A. blitoides, A. polygonoides, A. blitum, A. viridis, A. palmeri, A. spinosus* and parts of *A. tuberculatus* were compressed.
